# Does Loneliness Modulate Social Threat Detection in Daily Life?

**DOI:** 10.1093/geroni/igaf122.1684

**Published:** 2025-12-31

**Authors:** Sijing Shao, Emorie Beck, Zoe Hawks, Karina Van Bogart, Eileen Graham, Anthony Ong

**Affiliations:** FIU, Miami, Florida, United States; University of California, Davis, Davis, California, United States; Brooklyn Health, New York, New York, United States; Northwestern University, Chicago, Illinois, United States; Northwestern University, Evanston, Illinois, United States; Cornell University, Ithaca, New York, United States

## Abstract

Loneliness is theorized to heighten sensitivity to social threats, reinforcing maladaptive behaviors that perpetuate social isolation. This study used experience sampling methods (ESM) to examine how loneliness modulates social threat detection and social behaviors. A sample of 123 participants (46–74 years, M = 56.02, SD = 8.3, 69.1% female) completed five daily assessments over 20 days. A two-part Dynamic Structural Equation Model revealed small to medium autoregressive effects, suggesting both stability and within-person fluctuations in loneliness and perceived social threat. Concurrent effects showed a reciprocal relationship, where feeling lonely significantly increased the level of perceiving social threat at the same time point (est. effect = 0.335, 95% CrI [0.285, 0.0.386]), and vice versa. Greater fluctuations in loneliness were associated with greater fluctuations in perceived social threat (est. effect = 0.22, 95% CrI [0.14, 0.30]). However, trait loneliness did not significantly moderate these effects. Generalized linear mixed models indicated that within-person loneliness predicted lower self-disclosure (est. effect = -0.382, 95% CrI [-0.495, -0.274]), whereas trait loneliness showed no significant main effect. However, an interaction suggested that highly lonely individuals were less affected by momentary loneliness in their self-disclosure (est. effect = 0.144, 95% CrI [0.053, 0.237]). Interaction partner diversity had no effect on subsequent loneliness. Our findings reveal that loneliness heightens sensitivity to social threat, reducing social engagement and deepening social isolation. Understanding these dynamics can inform interventions targeting maladaptive cognitive and behavioral patterns associated with chronic loneliness.

